# Assessing Mitigation Translocation as a Tool to Reduce Human–great Horned owl Conflicts

**DOI:** 10.1007/s00267-025-02114-4

**Published:** 2025-01-11

**Authors:** Brian E. Washburn, Benjamin J. Massey, Alec C. Sonnek, Todd J. Pitlik

**Affiliations:** 1 United States Department of Agriculture, Animal Plant Health Inspection Service, Wildlife Services, National Wildlife Research Center, Fort Collins, CO USA; 2 United States Department of Agriculture, Animal Plant Health Inspection Service, Wildlife Services, Fort Collins, CO USA; 3 Present Address: United States Army Corps of Engineers, Pena Blanco, NM USA

**Keywords:** Airports, Bird strikes, *Bubo virginianus*, Great horned owls, Management, Predation

## Abstract

The great horned owl (*Bubo virginianus*) is a generalist predator that inhabits wide-ranging territories that are relatively stable throughout the year. These owls are also involved in a variety of human–owl conflicts, including killing of domestic poultry, predating colonially nesting seabirds and shorebirds, and pose a hazard to safe aircraft operations. Managing these conflict situations presents unique challenges as great horned owls are nocturnally active and occupy a wide range of habitats. We evaluated information about great horned owl collisions with civilian aircraft and found this is a contemporary and growing aviation safety issue. We conducted a study to determine whether a biological (e.g., age of the bird) and logistical factors (e.g., month and translocation distance) influenced the return rate of great horned owls following a mitigation translocation from 13 civil airports and three military airfields during 2013–2023. Great horned owls (*n* = 1,020) were live-captured, banded, and translocated various distances from the airfields which were then monitored for returning owls. We developed a set of candidate binomial-distributed generalized linear models [involving all possible subsets of three factors (age, month, and distance translocated) as well as interactions]. The return rate of translocated great horned owls was very low (i.e., 2.6%) and we found no evidence that these biological and logistical factors influenced great horned owl homing behavior. Management programs that use release sites 40 km from the conflict location and translocate individual owls only once would increase program efficacy by minimizing homing behavior and decreasing implementation costs.

## Introduction

The great horned owl (*Bubo virginianus*) has the most extensive distribution of any owl in the Americas (Duncan 2003; Artuso et al. 2022). Great horned owls are abundant in forested, grassland, desert, and anthropogenically altered (e.g., suburban and urban) landscapes (Artuso et al. 2022). They are generalist predators with one of the most diverse prey profiles of all North American raptors (Duncan 2003). Great horned owls use a ‘perch-and-pounce’ foraging strategy and are active nocturnally. These owls are non-migratory and breeding adults hold territories throughout the year (Holt 1996; Rohner 1996).

Human-wildlife conflicts occur worldwide and encompass a broad spectrum of situations where humans and/or wildlife directly or indirectly impact (or are perceived as impacting) one another, often involving human health, safety, and well-being, agricultural commodities, natural resources (especially those of cultural or ecological importance), infrastructure, or recreation (Peterson et al. 2010; Nyhus 2016, Conover and Conover 2022). Great horned owls are involved in many human-raptor conflicts, including depredation of backyard domestic poultry (Washburn 2016), predation upon colonial waterbirds and nesting shorebirds and seabirds (Catlin et al. 2011; Pollet and Shutler 2019; Serré et al. 2023; Stantial et al. 2024), and human health and safety issues involving collisions with vehicles (e.g., cars, aircraft; Franson and Little 1996; Bishop and Brogan 2013; Linnell and Washburn 2018). Also, great horned owls are formidable predators that can kill and eat other raptors, such as peregrine falcons (*Falco peregrinus*; Cade et al. 1989; Craig and Enderson 2004; Dzialak et al. 2005), osprey (*Pandion haliaetus*; Ewins 1997; Artuso et al. 2022), and swallow-tailed kites *(Elanoides forficatus*; Coulson et al. 2008). Such predation events can be costly to reintroduction programs with the goal of increasing raptor populations or expanding the range of those species.

Of particular importance, great horned owls pose a hazard to safe aircraft operations at civilian airports and military airfields throughout North America. Among the 13 North American owl species that have been involved in aircraft strikes, great horned owls were the third most frequently struck species (Linnell and Washburn 2018). Great horned owls were ranked as the 30th riskiest species in a study of the economic risk of bird strikes with civilian aircraft associated with 76 bird species (DeVault et al. 2018). Similarly, Pfieffer et al., (2018) quantified and ranked avian hazards to military aircraft (more specifically from the U.S. Air Force and U.S. Navy) and listed great horned owls as the 24th most hazardous among the more than 100 bird species evaluated. A better understanding of great horned owl collisions with aircraft is needed to allow for the development of effective resolutions to this important issue.

We queried the Federal Aviation Administration’s National Wildlife Strike Database and found that during 1990–2023, 407 great horned owl-aircraft collisions (an average of 12.0 ± 1.7 SE annually) were reported. Aircraft collisions involving this species increased by 2500% during the same time period. Great horned owl strikes occurred throughout the year, but 53% of these events occurred from September through December. Higher owl-aircraft strike rates during fall and early winter might be related to increased airfield use by great horned owls due to juvenile natal dispersal or adult post-breeding dispersal of adults. Among the 390 great horned owl strikes for which the specific geographic location could be determined, the states with the highest number of reported events were Colorado (25%), California (7%), Missouri (7%), and Texas (6%). Great horned owl strikes were also reported in 39 other states, although 15 or less strikes were reported per state. Forty-six of the great horned owl collisions with aircraft resulted in damage to the aircraft; the estimated total cost was more than $7.8 million USD. Almost all of these events resulted in mortality of the owls involved.

Many tools, such as non-lethal hazing with pyrotechnics (Marsh et al. 1991), are valuable for managing avian hazards at airports during the daytime; however, these methods are not practical during the night when nocturnal species (e.g., great horned owls) are active. Consequently, few options are available for managing the risks to safe aircraft operations posed by great horned owls or to alleviate great horned owl predation upon threatened and endangered shorebirds. Mitigation translocation (i.e., the live-capture and translocation of individuals away from the location where the conflict is occurring) and lethal removal of problematic individuals (Baxter and Allan 2008) are arguably the most practical methods available to resolve such conflicts.

Mitigation translocation is commonly used in a wide array of human-wildlife conflict situations (Fischer, Lindenmeyer 2000; Sullivan et al. 2015). Translocating problematic wildlife is often perceived as a panacea for resolving human-wildlife conflicts and a large proportion of the public favors non-lethal management actions over lethal removals (Massei et al. 2010). Unfortunately, management programs involving mitigation translocations are typically conducted without any monitoring or means of determining whether actions were successful (Germano et al. 2015; Bradley et al. 2022).

No published information is available regarding the efficacy of great horned owl hazard management programs for reducing the abundance on and use of airfields by these birds and thus reducing the frequency of owl-aircraft collisions within airport environments. Effective, publicly-acceptable methods to reduce the hazards posed by great horned owls to aviation safety are needed. We conducted a study to increase our understanding of using mitigation translocation as a non-lethal wildlife damage management tool for reducing the frequency of great horned owl–aircraft collisions.

Based on our understanding of owl seasonal movements and land cover use (Rohner 1996; Artuso et al. 2022), we hypothesized that a biological factor (i.e., age of individual owls) and two logistical factors related to mitigation translocation events (i.e., month when the translocation occurred and the distance of the release site away from an airfield) would influence the frequency of homing behavior in great horned owls. We predicted that older birds would have higher return rates than younger birds and that translocating individual owls farther away from the capture location would reduce return rates due to increased energetic costs of flying longer distances. The objectives of our study were to: (1) determine return rates of great horned owls following a mitigation translocation, and (2) evaluate factors that might influence the return rate of translocated great horned owls.

## Methods

### Study Areas

We conducted this study within the states of California, Colorado, Iowa, and Missouri. Non-lethal management efforts regarding great horned owls were conducted at 13 civilian airports and three military airfields (Table 1).

**Table 1:** Civil airports and military airfields where great horned owls were live-captured and translocated, 2013–2023

Civil Airport or Military Airfield	State	Latitude	Longitude
John Wayne/Orange County Airport	California	33.675662	–117.868233
Joint Forces Training Base Los Alamitos	California	33.790030	–118.051420
Long Beach Airport	California	33.817930	–118.151891
Los Angeles International Airport	California	33.942496	–118.408049
Naval Base Ventura County	California	34.113405	–119.106012
Riverside Municipal Airport	California	33.950544	–117.445930
Van Nuys Airport	California	34.209806	–118.489972
Denver International Airport	Colorado	39.856350	–104.676400
Des Moines International Airport	Iowa	41.532921	–93.649081
Sioux Gateway Airport	Iowa	42.400890	–96.378731
Charles B. Wheeler Downtown Airport	Missouri	39.125302	–94.587330
Kansas City International Airport	Missouri	39.301409	–94.710454
Rosecrans Memorial Airport	Missouri	39.770363	–94.903233
Spirit of St. Louis Airport	Missouri	38.663473	–90.644578
St. Louis Lambert International Airport	Missouri	38.749940	–90.374819
Whiteman Air Force Base	Missouri	38.728996	−93.560551

The California study area encompasses the southern California geographic region, including the coastal lowland area of the Los Angeles Basin, the foothills of the Santa Ana Mountains and the San Jaquin Hills, and extending into the Mojave desert. This area has a Mediterranean climate, characterized by an average daily temperature during summer of 27° C and 20° C during winter. Mean annual precipitation is 305 mm per year, most of which falls during the winter months (primarily February).

The Colorado study area encompassed large portions of the high plains of Colorado, the Front Range of the Rocky Mountain chain, and some areas within the Rocky Mountains themselves. The climate in this study area is relatively cool and dry, with large daily and seasonal changes in temperature. This study area has an average daily temperature during summer of 30° C, an average daily temperature of –9° C during winter, and mean annual precipitation is 463 mm per year (most of which occurs as rain and storms during March through August).

The midwestern study area (comprising the states of Missouri and Iowa) encompassed the northern plains (e.g., prairie region), the Osage plains, and parts of the Ozark Plateau of Missouri and the flat plains of Iowa. This area has a continental type of climate marked by strong seasonality. Moderately cold winters and hot, humid summers are characteristic of this part of the Midwest, with an average daily temperature during summer of 31° C and –6° C during winter. Mean annual precipitation is 1524 mm per year, with three times as much precipitation falling as rain than snow.

### Live-capture and Handling

During 6 September 2013–21 March 2023, United States Department of Agriculture-Wildlife Services (USDA WS) personnel used Swedish goshawk traps (Bub 1991; Bloom et al. 2007) to live-capture great horned owls that were presenting a hazard to aircraft at 13 civilian airports and 3 military airfields in the states of California, Colorado, and Missouri.

All live-captured great horned owls were assigned to age classes based on plumage and feather molt (Pyle 1997; Pyle 2008). We used the age classification system employed by the U.S. Geological Survey (USGS) Bird Banding Laboratory. Hatching-year (HY) birds were between 6 weeks and 9 months old at the time of capture, second-year (SY) birds were between 9 and 21 months of age, and after-second-year (ASY) birds were ≥22 months of age. Although great horned owls exhibit sexual dimorphism, accurately placing individuals into sex classes is not possible without genetic testing. Each live-captured great horned owl was banded with a standard USGS aluminum leg band.

### Mitigation Translocations

Using a stratified random selection process to ensure relatively equal sample sizes, we assigned each owl to one of four pre-selected release location distance. Translocated individuals were released at locations that were 40 km, 80 km, 121 km, and 161 km from the airport where the bird was live-captured. All great horned owls were held for <48 h (from time of capture to release). We kept all owls under climate-controlled conditions, in individual cages, and with minimal human disturbance while in captivity. Release sites selected for use in this study were typically in an eastern or western direction from the airport or airbase. Release sites consisted of urban parks, golf courses, state wildlife management areas, and other greenspaces within both urbanized and rural landscapes. We ensured that all release sites located away from major interstate highways and airports.

### Monitoring for Great Horned Owl Returns

We define a great horned owl return as any situation where an individual owl was resighted (e.g., visually observed) or recovered (e.g., re-captured or found dead) at an airport or military airfield following a mitigation translocation event. Live-trapping activities for great horned owls were conducted on the airports and airbases from September 2013 (the start of this study) to March 2024 (one year after the marking phases of the project ended in March 2023). Biologists examined all great horned owls that were recaptured for the presence of a federal bird band (if possible) and recorded the identity of all known individuals in addition to other pertinent information (e.g., date, time, location).

Great horned owls that returned to a civilian airport or military airfield following a mitigation translocation were managed according to predetermined, wildlife-damage management actions outlined within the facility’s airport wildlife hazard management plan. More specifically, when these individuals were re-captured they were translocated away from the aviation facility for a second time to a randomly selected distance.

### Statistical Analyses

Great horned owl return (i.e., following the first translocation event) was a binary response variable, with 0 representing individuals that were not resighted or recaptured on an airport or airbase following the first translocation event and 1 representing those that returned to an airport or airbase at some point in time. Initially, we used a generalized linear mixed model approach and included the location (i.e., airport) where an owl was live-captured as a random factor in all models. However, the random effect variance was essentially ‘zero’ and thus we removed the random effect from all models for further analyses. We developed a set of candidate generalized linear mixed models (binomial distribution) fit with Laplace estimation [involving all possible subsets of three factors (age, month, and distance translocated) as well as interactions].

We used Akaike’s Information Criterion (AIC; Burnham and Anderson 2002) and whether explanatory coefficients in the selected model(s) were informative (i.e., 95% CIs did not overlap 0; Arnold 2010) to evaluate the great horned owl return models. Any models within 2 ΔAIC units of the top ranked model we considered to be competitive (Symonds and Moussalli 2011). We used Program R Version 4.3.3 (R Core Team 2024) and the R package lme4 (Bates et al. 2015) to evaluate the generalized linear models.

We used a *G*-test for independence test (Zar 1999) to compare the percentage of great horned owls that returned following the first mitigation translocation to the percentage of great horned owls that returned following a second mitigation translocation.

We determined the number of days from the translocation date to the first resight or recovery of the bird for each individual great horned owl that returned to an airport or airbase and defined this period as days to return. Prior to conducting analyses, we used Shapiro-Wilk tests (to ensure normality) and Levene’s tests (to ensure equality of variances) for dependent variables (Neter et al. 1990). We used one-way analysis of variance (ANOVA) to compare the days to return among the three age classes of owls and among the four translocation distances (Neter et al. 1990; Zar 1999).

## Result**s**

### Mitigation Translocations

**Fig. 1:**
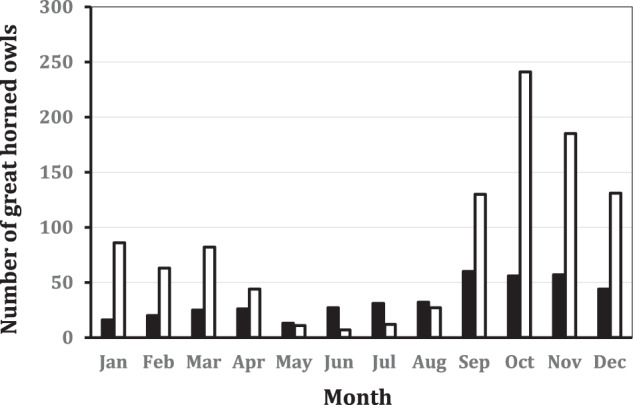
Monthly distribution of great horned owl collisions with U.S. civilian aircraft (annually) within the USA reported to the Federal Aviation Administration from 1990 to 2023 (*n* = 407; black bars) and the monthly distribution of mitigation translocations of great horned owls from a civil airport or military airbase in California, Colorado, Iowa, or Missouri, USA, 2013–2023 (*n* = 1020; white bars)

We live-captured 1020 individual great horned owls and conducted 1047 mitigation translocation events (some owls were translocated twice) during 2013–2023. The initial mitigation translocations involved 375 HY, 228 SY, and 416 ASY great horned owls, respectively. We conducted 218, 253, 295, and 253 great horned owl initial mitigation translocations to the 40 km, 80 km. 121 km, and 161 km distances, respectively. Mitigation translocations of great horned owls occurred during all months of the year, but 67% of all events occurred from September through December (Fig. 1). Interestingly, the number of great horned owl strikes per month was correlated (Pearson’s Correlation: *r* = 0.78, 12 df, *P* = 0.003) with the number of great horned owl mitigation translocations conducted per month.

### Return Rates

**Fig. 2:**
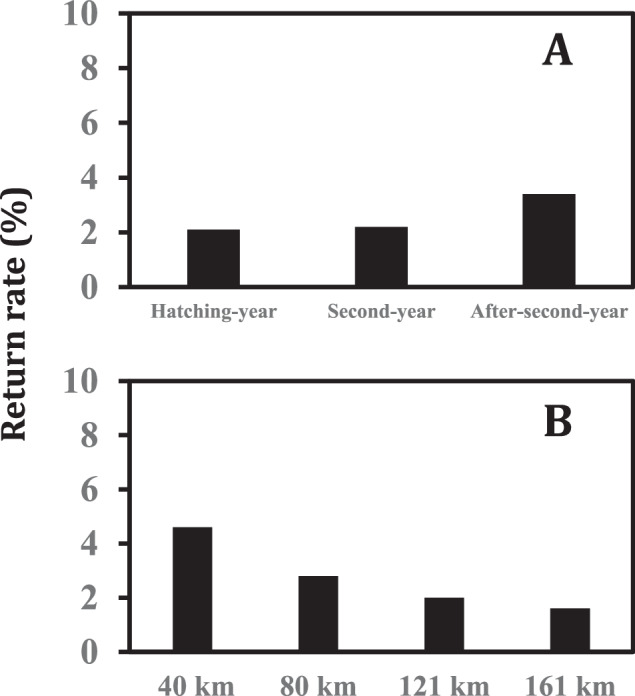
Return rates (%) of great horned owls following a mitigation translocation as influenced by **A** the age of the birds and **B** the distance they were translocated from a civil airport or military airbase in California, Colorado, Iowa, or Missouri, USA, (2013–2023)

Overall, we documented that only 2.6% of the great horned owls exhibited homing behavior and returned to an airport or military airfield following a mitigation translocation. Homing behavior of great horned owls (as assessed by return rates) was below 5% for all three age classes and for the four translocation distances that owls were taken from the airfields (Fig. 2).

The top ranked model, with approximately one third of the Akaike weight (*w*_*i*_) = 0.32, included month as a fixed factor (Table 2). Two other models were competitive, the null model and the month + age (additive) model (Table 2). However, the 95% CIs for both month (*β* = –1.34, SE = 1.18, Lower 95% CI = –3.65, upper 95% CI = 0.97) and age (*β* = –0.42, SE = 0.49, Lower 95% CI = –1.38, upper 95% CI = 0.54) encompassed ‘0’ (Table 3). Thus, it appears none of the fixed factors we examined influenced the return rates of great horned owls following a mitigation translocation.

**Table 2:** Top bionomial generalized linear models (binomial distribution), ranked by Akaike’s Information Criterion (AIC), predicting great horned owl returns following a translocation event (*n* = 1020) from a civil airport or military airfield in California, Colorado, Iowa, or Missouri, USA, 2013–2023

Model	*K*^a^	LL^b^	AIC	∆AIC^c^	*w*_*i*_^d^	Cumulative *w*_*i*_
Month	12	−113.26	250.52	0.00	0.32	0.32
Null	1	−124.70	251.39	0.87	0.21	0.52
Age + Month	14	–111.91	251.82	1.30	0.17	0.69
Distance	4	–122.51	253.03	2.51	0.09	0.78
Month + Distance	15	–111.60	253.20	2.67	0.08	0.87
Age	3	–124.02	254.03	3.51	0.06	0.92
Month + Distance + Age	17	–110.39	254.77	4.25	0.04	0.96
Distance + Age	6	–121.74	255.47	4.95	0.03	0.99
Distance*Age	12	−116.36	256.72	6.20	0.01	1.00

^a^ Number of parameters in model

^b^ Log likelihood

^c^ Difference in AIC compared with lowest AIC model

^d^ Model weight

**Table 3:** Parameter estimates with unconditional standard errors (SE) and 95% confidence intervals (lower [LCL] and upper [UCL]) for great horned owl returns following a translocation event (*n* = 1020) from a civil airport or military airfield California, Colorado, Iowa, or Missouri, USA, 2013–2023

Parameter	Estimate	SE	LCL	UCL
Intercept	−2.14	0.65	−0.87	−3.41
Month	–1.34	1.18	−3.65	0.97
Age	–0.42	0.49	−1.38	0.54

### Known Fates of Translocated Owls

**Fig. 3:**
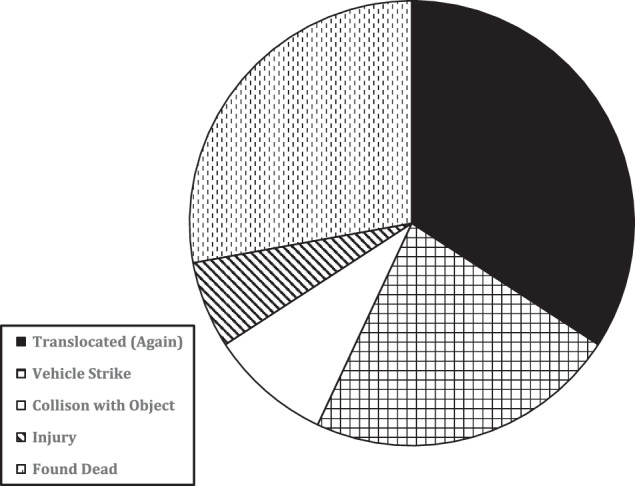
Distribution of known fate outcomes for great horned owls (*n* = 79) following a mitigation translocation from a civil airport or military airbase in California, Colorado, Iowa, or Missouri, USA, 2013–2023

Approximately 92% of translocated great horned owls (*n* = 1020) were not observed or recovered post-release after the initial mitigation translocation and we consider these individuals to be of unknown fate. We were able to determine the known fate for 79 great horned owls, including 27 which returned to an airport or airbase following the first mitigation translocation event (Fig. 3). All great horned owls returned to the same airport or airbase from which they were translocated. Upon their return from the mitigation translocation, the 27 great horned owls were taken on a second mitigation translocation away from the airport. Interestingly, the proportion of great horned owls that returned to an airport following the first mitigation translocation (2.6%) was less (*G* = 75.44, *P* < 0.0001) than the proportion of owls returning after a second mitigation translocation (40.7%).

Two-thirds (66%) of the great horned owls with known fates were resighted or recovered by the general public and reported to the U.S.G.S. Bird Banding Laboratory. All of the public resighting reports suggest that owls were involved in collisions with anthropogenic objects, including vehicles (which accounted for at least 35% of the known mortalities).

### Days to Return

We found no difference (*F*_2,26_ = 0.29, *P* = 0.75) in the days to return among the three age classes of great horned owls (Table 4). In addition, great horned owls translocated to the four distances returned to the airports during the same amount of time (*F*_3,26_ = 1.35, *P* = 0.28; Table 4). Thus, the number of days it took translocated great horned owls to return to an airfield following a mitigation translocation was not influenced by either age of the birds or the distance they were translocated.

**Table 4:** Days to return for great horned owls following a translocation event (*n* = 27) from a civil airport or military airfield in California, Colorado, Iowa or Missouri, USA, 2013–2023

Parameter	*N*	$\bar{x}$	SE	Min.	Max.
Age Classes					
Hatching–year^a^	8	384.0	109.4	48	804
Second–year^b^	5	265.0	116.3	12	616
After–second–year^c^	14	414.4	116.5	7	1,621
Distance Translocated					
40 km	10	313.1	93.7	23	834
80 km	7	328.3	109.4	7	745
121 km	6	321.0	133.6	12	804
161 km	4	710.8	308.4	312	1,621

^a^Hatching–year birds were between 6 weeks and 9 months of age

^b^Second–year birds were between 9 and 21 months of age

^c^After–second–year birds were ≥22 months of age

## Discussion

Despite being recognized as posing a hazard to aviation safety, information on great horned owl management programs has been lacking. Great horned owls colliding with civilian aircraft is a contemporary and growing aviation safety issue within the United States. Although great horned owl collisions with aircraft occurred in most U.S. states, Colorado, California, Missouri, and Texas had the highest frequencies of such events.

Homing behavior of great horned owls after a mitigation translocation was limited and not influenced by the biological or logistical factors examined during our study. Interestingly, homing behavior (as determined by return rates) of great horned owls were considerably lower that what has been found for other raptors [e.g., burrowing owls (*Athene cunicularia*; Santos et al. 2023), snowy owls (*Bubo scandiacus*; McCabe et al. 2022), red-tailed hawks (*Buteo jamaicensis*; Pullins et al. 2018; Washburn et al. 2021), and vultures (Galvão Novaes et al. 2020)]. The return rate of great horned owls in our study (2.6%) was consistent with other efforts examining great horned owl mitigation translocation from airports in southern California (2.1%; Biteman et al. 2018) and at many airports across the USA (3.4%; Schafer and Washburn 2016). We believe all of these estimates are conservative, as detection and identification of marked and unmarked nocturnally active owls can be very challenging and thus it is likely that not all owls that returned following a mitigation translocation were observed or recaptured.

Interestingly, great horned owls translocated for a second time were more than 15 times more likely to return compared to after the initial translocation event. This finding is consistent with homing behavior patterns observed in red-tailed hawks and American kestrels (*Falco sparverius*) following a mitigation translocation. Pullins et al. (2018) found that the odds of a red-tailed hawk returning to an airport increased by a factor of 12 for each subsequent translocation event. Similarly, Washburn (2024) found that compared to an initial mitigation translocation, American kestrels were more than 10 times more likely to return following a second mitigation translocation.

Relatively few (7.4%) great horned owls that returned to an airfield following a mitigation translocation did so within a month. Approximately 40% of the returning individuals did so after (apparently) being elsewhere for at least a year (and for one owl up to four years). Alternatively, it is possible that these owls returned to the airport environment but did not get recaptured until a later date. Interestingly, on average great horned owls took three times longer than red-tailed hawks (Pullins et al. 2018) and 9 times longer than American kestrels (Washburn 2024) to return to an airport following a mitigation translocation. Thus, not only did few great horned owls return but those that did took considerably longer to do so, consequently reducing the hazards to aviation posed by these individual owls.

Over half of the great horned owl collisions with aircraft and two-thirds of the mitigation translocation events occurred during fall months, suggesting much of the airfield use by great horned owls is occurring during this time of year. Seasonally higher levels of great horned owl abundance and activity on airfields during these months might be associated with juvenile dispersal (Rohner 1996), territory searching and establishment activities by pre-breeders and adults (Artuso et al. 2022), or seasonally abundant prey resources. Future research studying the season habitat use and movement patterns of great horned owls using airport environments would provide valuable insights.

The abundance of hazardous wildlife, as well as the specific behaviors exhibited by those species, within the airfield environment is an important factor related to the likelihood of wildlife-aircraft collisions (DeVault et al. 2013, 2018). However, determining the presence and abundance of nocturnally active species, such as great horned owls, within active airfield environments can be particularly challenging as traditional auditory survey methods (Anderson 2007; Reynolds et al. 2021) would be impractical and non-territorial ‘floaters’ do not respond to conspecific calls (Rohner 1996). Novel means of detecting and counting great horned owls on airports or in other conflict situations are needed. Such methods would allow a better understand of how the abundance and behavior of great horned owls (and other wildlife hazardous to aviation) within airport environments influences the potential for owl-aircraft collisions and thus lead forward to effective solutions.

Great horned owls are generalist avian predators that use a very diverse prey base (Artuso et al. 2020). However, numerous studies have shown that mammals (e.g., lagomorphs and small mammals) comprise the largest proportion of great horned owl diets in most areas and that owl prey selection is typically based on the abundance or availability of specific mammalian species within a given landscape (Zimmerman et al. 1996; Donázar et al. 1989; Cromrich et al. 2002). Managing populations of lagomorphs and/or small mammals to reduce the attractiveness of airfields to foraging great horned owls might be a valuable component of an integrated hazard reduction program in some situations (Washburn et al. 2011; Witmer 2011) but this remains unstudied.

Consistent with our findings, many known great horned owl mortalities have an anthropogenic influence; major contemporary challenges faced by great horned owl populations include collisions with vehicles (e.g., automobiles, aircraft), electrocutions, and secondary effects of rodenticides (Franson and Little 1996; Houston and Francis 1996; Smith et al. 2018). However, other research has shown that owls killed by human-related causes are more likely to be discovered than those that die of natural factors (e.g., disease, starvation, predation) far from human development (Franson and Little 1996; Houston and Francis 1996).

Although our study is focused on great horned owl conflicts associated with civilian airports and military airfields, the findings have direct implications for the management of other human-owl conflicts. Great horned owls often prey upon federally threatened and endangered shorebirds, such as piping plovers (*Charadrius melodus*) and roseate terns (*Sterna dougalliillii*), typically while these shorebirds are nesting (Murphy et al. 2003; Stantial et al. 2024). Such conflicts typically involve only a few individual great horned owls, but the impacts of their predation can substantially decrease productivity (Catlin et al. 2011; Stantial et al. 2024). Great horned owl predation on other raptors, such as peregrine falcons, ospreys, and swallow-tailed kites, can have negative consequences for management actions (e.g., hacking programs) to enhance existing populations or expand the range of those species (Cade et al. 1989; Dzialak et al. 2005). Managing great horned owls using mitigation translocation might be a valuable option to resolve such conflicts. We suggest studies evaluating the efficacy of management programs (including mitigation translocation or other tools) to reduce non-aviation related human-owl conflicts represents an area of valuable and important future research.

The recent development of highly accurate GPS-capable transmitters small enough to be used on great horned owls (although the nocturnal activity of this species creates additional challenges) provides excellent opportunities to advance our understanding of both the basic ecology of the species and what factors are important in developing science-based and effective management programs. This technology could be particularly useful for increasing our understanding of great horned owl seasonal movements and habitat use within highly urbanized landscapes. Also, increasing our understanding of how and when owls use locations where vehicle-collision mortality is substantial (e.g., airports, highway corridors) represents an important area of future research. Determining the survival, post-translocation movement patterns, and habitat use of translocated great horned owls would be a very important area for future research and would provide highly valuable information for the development of efficacious management programs.

Management decisions on specific methods and practices to alleviate conflicts between humans and great horned owls involves a complex set of biological and social variables [e.g., the biology of the species involved (this study; Côte and Sutherland 1997), direct economic costs of management actions, hidden logistical costs (Massei et al. 2010), personal and corporate liability of wildlife strikes (Dale 2009), and public perception of the management program in an increasingly social media-focused world (Washburn 2018)]. This study represents an important step in providing a scientific foundation for the development of efficacious management programs to reduce the frequency and negative impacts of these conflicts.

## Conclusions

Mitigation translocation of raptors (such as great horned owls) is an important component of integrated wildlife damage mitigation programs at many civilian airports and military bases. Based on our findings, we recommend that to reduce the potential for great horned owl conflicts and consequently maximizing the benefits of program resources, damage management programs should consider live-capturing and translocating great horned owls to a distance of at least 40 km from the location of the problem (e.g., an airport) and translocated individual owls only one time. In addition, other potential factors might influence the behavior of great horned owls after a mitigation translocation and thus should be investigated.

## Data Availability

Data for this study have been archived and are available from the USDA/APHIS/Wildlife Services National Wildlife Research Center.
